# Se_2_Mo_10_V_3_, a heteropoly compound containing selenium, inhibits tumor growth

**DOI:** 10.18632/oncotarget.18909

**Published:** 2017-07-01

**Authors:** Hong-Ning Zhang, Wei-Li Feng, Chun-Na An, Wen-Guang Li

**Affiliations:** ^1^ Department of Pharmacology, School of Basic Medical Sciences, Capital Medical University, Beijing 100069, China; ^2^ Department of Pharmacology, School of Basic Medical Sciences, Qinghai University, Xining 810001, China; ^3^ Department of Pharmacology, School of Basic Medical Sciences, North China University of Science and Technology, Tangshan 063009, China; ^4^ Department of Pharmacology, School of Basic Medical Sciences, Lanzhou University, Lanzhou 730000, China

**Keywords:** heteropoly compound containing selenium, Se_2_Mo_10_V_3_, anti-tumor, apoptosis, NF-κB/IκBα

## Abstract

Selenium compounds have strong anti-tumor effects and are well-tolerated. We examined the anti-tumor effects of (NH_4_)_2_H_15_Se_2_^VI^Mo_10_V_3_O_52_·2H_2_O (Se_2_Mo_10_V_3)_, a heteropoly compound containing selenium. Se_2_Mo_10_V_3_ inhibited proliferation in K562 cells with a half-maximal inhibitory concentration of 78.72±2.82 mg/L after 48 h and 24.94±0.88 mg/L after 72 h. Typical apoptotic morphologies were also observed in K562 cells treated with Se_2_Mo_10_V_3_, as were increased intracellular levels of Ca^2+^, Mg^2+^, H^+^, and reactive oxygen species, and decreased mitochondrial membrane potential. In addition, Se_2_Mo_10_V_3_ treatment triggered cytochrome C release and inhibited IκBα degradation and NF-κB translocation. *In vivo* experiments revealed that 5 or 10 mg/kg Se_2_Mo_10_V_3_ inhibited the growth of sarcoma 180 and hepatoma 22 xenograft tumors. These results indicate that Se_2_Mo_10_V_3_ inhibits tumor growth both *in vitro* and *in vivo* and induces apoptosis in K562 cells, possibly by inhibiting the NF-κB/IκBα pathway.

## INTRODUCTION

Selenium, an essential non-metal trace element [1], is crucial to the function of several enzymes, including glutathioneperoxidases and thioredoxin reductases [2]. Selenium deficiency is related to tumorigenesis, and selenium recruitment reduces the risk of cancer in humans [3, 4]. Furthermore, selenium compounds can prevent tumor formation [5], mainly by inducing apoptosis [6]. For example, Yang and Wang found that Na_5_SeV_5_O_18_.H_20_, a heteropoly compound containing selenium, had anti-tumor effects both *in vitro* and *in vivo* [7]. When used as therapeutic agents in tumor patients, selenium compounds are well-tolerated [8].

The possible anti-tumor effects and related underlying mechanisms for another selenium-containing heteropoly compound, (NH_4_)_2_H_15_Se_2_^VI^Mo_10_V_3_O_52_·2H_2_O (abbreviation: Se_2_Mo_10_V_3_), have not yet been studied. Here, we examined the anti-tumor effects of Se_2_Mo_10_V_3_ and the mechanisms underlying those effects.

## RESULTS

### Se_2_Mo_10_V_3_ inhibited proliferation in K562 cells

**Figure 1:**
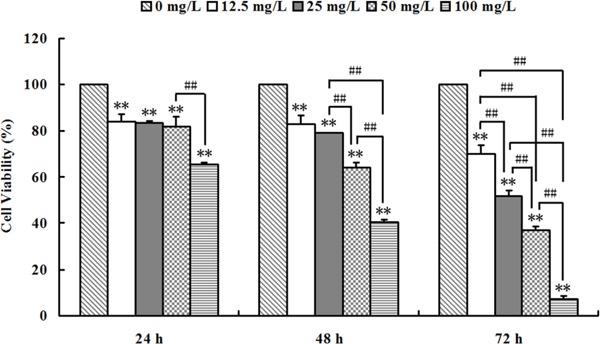
Se_2_Mo_10_V_3_ inhibited proliferation in K562 cells in an MTT assay Data are expressed as means ± S.D. of three independent experiments. *P<0.05, ^*^P<0.01 versus control cells.

As shown in Figure 1, Se_2_Mo_10_V_3_ (12.5-100 mg/L) inhibited proliferation in K562 cells. The inhibitory rates for the 12.5, 25, 50, and 100 mg/L doses were 15.91±2.94, 16.64±0.97, 17.93±3.92, and 34.65±0.73% after 24 h (all *P*<0.05), 17.02±3.77, 20.78±0.15, 35.79±1.83, and 59.51±1.16% after 48 h (all *P*<0.05), and 30.12±3.76, 48.07±2.16, 62.93±1.63, and 92.77±1.51% after 72 h (all *P*<0.01), respectively, with IC50 values of >100 mg/L after 24 h, 78.72±2.82 mg/L after 48 h, and 24.94±0.88 mg/L after 72 h. The inhibitory rate in control cells was set to 0. In positive control K562 cells treated with carboplatin (12.5-100 mg/L), IC50 values were >100 mg/L after 24 h, 52.39 mg/L after 48 h, and 25.00 mg/L after 72 h. Similar inhibitory effects were observed in A549, CNE, SPCA-1, and Lewis cells after Se_2_Mo_10_V_3_ treatment (data not shown).

**Figure 2:**
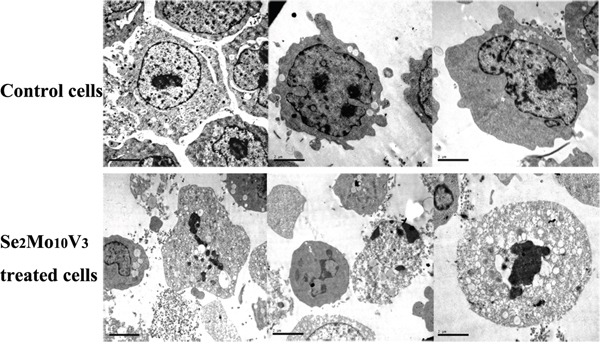
50 mg/L Se2Mo10V3 induced apoptosis in K562 cells in a TEM expreiment Scale = 2.00 μm. Data are representative of three independent experiments.

### Se_2_Mo_10_V_3_ induced apoptotic morphology in K562 cells

**Figure 3:**
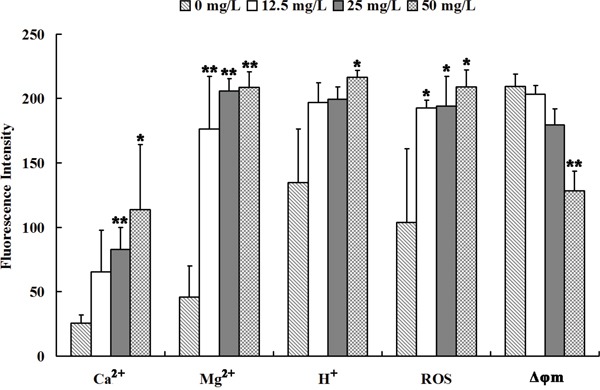
Se2Mo10V3 increased Ca2+, Mg2+, H+, and ROS levels and decreased Δφm in K562 cells in a CLSM experiment Data are expressed as means ± S.D. of three independent experiments. *P<0.05, ^*^P<0.01 versus control cells.

As shown in Figure 3, typical indicators of apoptosis, such as chromatin fragmentation, condensation and margination, cytoplasm vacuoles, and disappearance of cytolemma microvilli, were observed to a greater extent in K562 cells treated with 50 mg/L Se_2_Mo_10_V_3_ than in control cells, which were characterized by normal chromatin, uniform cytoplasm, the presence of cytolemma microvilli, and diastolic cell bodies.

### Se_2_Mo_10_V_3_ increased Ca^2+^, Mg^2+^, H^+^, and ROS levels and decreased Δφ_m_ in K562 cells

**Figure 4:**
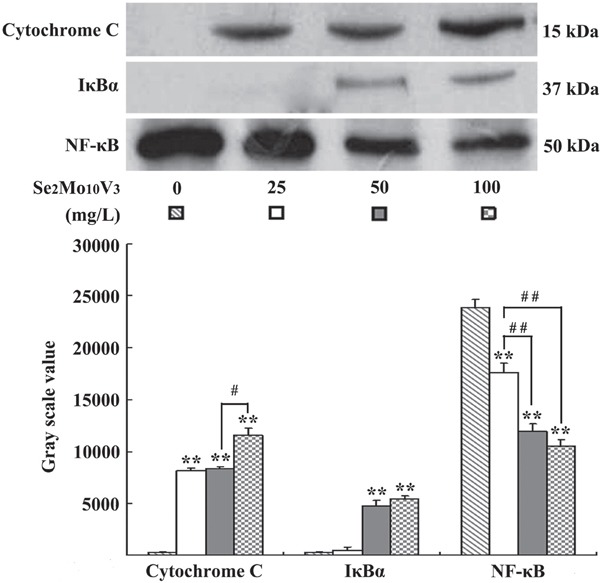
Se_2_Mo_10_V_3_ promoted cytoplasmic cytochrome C level, inhibited cytoplasmic IκBα degradation and reduced nuclear NF-κB level in K562 cells in a Western blotting experiment. Data are expressed as means ± S.D. of three independent experiments. *P<0.05, **P<0.01 versus control cells.

The results of CLSM are shown in Figure 4. Intracellular Ca^2+^ levels increased after treatment with 25 or 50 mg/L Se_2_Mo_10_V_3_ (both *P*<0.05). Intracellular Mg^2+^ levels also increased after treatment with 12.5, 25, or 50 mg/L Se_2_Mo_10_V_3_ (all *P*<0.01). Intracellular H^+^ levels also increased, indicating that intracellular pH decreased, after treatment with 50 mg/L Se_2_Mo_10_V_3_ (*P*<0.05). In addition, intracellular ROS levels increased after treatment with 12.5, 25, or 50 mg/L Se_2_Mo_10_V_3_ (all *P*<0.05). Finally, Δ*φ*_m_ was markedly reduced after treatment with 50 mg/L Se_2_Mo_10_V_3_ (*P*<0.01).

### Se_2_Mo_10_V_3_ promoted cytoplasmic cytochrome C level, inhibited cytoplasmic IκBα degradation and reduced nuclear NF-κB level in K562 cells

**Figure 5:**
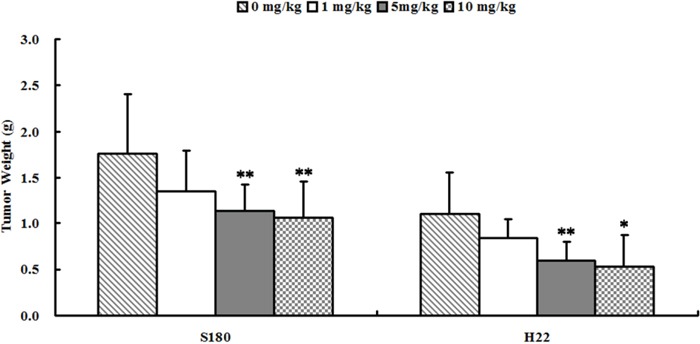
Se_2_Mo_10_V_3_ inhibited the growth of S180 and H22 xenografts *in vivo* Data are expressed as means ± S.D. (n=10). *P<0.05, **P<0.01 versus control tumor-bearing mice.

Western blotting results are shown in Figure 5. Cytoplasmic cytochrome C levels were increased in cells treated with 25, 50, or 100 mg/L Se_2_Mo_10_V_3_ (all *P*<0.01), which indicated that the release of cytochrome C from the mitochondria into the cytoplasm was increased. Cytoplasmic IκBα degradations were decreased in cells treated with 50 or 100 mg/L, but not with 25 mg/L Se_2_Mo_10_V_3_ (both *P*<0.01), and nuclear NF-κB levels were reduced in cells treated with 25, 50, or 100 mg/L Se_2_Mo_10_V_3_ (all *P*<0.01), which indicated that the translocation of NF-κB from the cytoplasm to the nucleus was inhibited.

### Se_2_Mo_10_V_3_ inhibited S180 and H22 cell growth *in vivo*

As shown in Figure 2, 5 or 10 mg/kg Se_2_Mo_10_V_3_ (i.g.) inhibited the growth of S180 cells by 35.6 and 39.4%, respectively (all *P*<0.01). In S180 tumor-bearing mice, tumor weights were 1.762±0.642, 1.351±0.442, 1.135±0.275, and 1.067±0.379 g after treatment with 0, 1, 5, and 10 mg/kg Se_2_Mo_10_V_3_, respectively. Five or 10 mg/kg Se_2_Mo_10_V_3_ (i.g.) similarly inhibited the growth by 46.4 and 52.6%, respectively, in H22 cells (both *P*<0.05). In H22 tumor-bearing mice, tumor weights were 1.113±0.447, 0.851±0.203, 0.597±0.212, and 0.528±0.351 g after treatment with 0, 1, 5, and 10 mg/kg Se_2_Mo_10_V_3_, respectively.

## DISCUSSION

In this study, we demonstrated that Se_2_Mo_10_V_3_, a heteropoly compound containing selenium, had anti-tumor effects both *in vitro* and *in vivo*. Furthermore, we explored the mechanisms underlying its anti-tumor effects in K562 cells.

Apoptosis, a genetically controlled mechanism essential for the maintenance of tissue homeostasis and proper development, is crucial to the anti-tumor effects of many chemotherapeutics [9]. Here, we observed typical apoptotic morphology, including chromatin fragmentation, condensation and margination, cytoplasm vacuoles, and the absence of cytolemma microvilli, in Se_2_Mo_10_V_3_-treated K562 cells here. Ca^2+^ and Mg^2+^ are key intracellular messengers that play important roles in the induction of apoptosis. Increases in cytosolic Ca^2+^ and Mg^2+^ levels have been linked to the activation of a Ca^2+^/Mg^2+^ dependent endonuclease which cleaves DNA to generate nucleosomal fragments (180-200 bp) during apoptosis [10, 11]. Here, we confirmed that Se_2_Mo_10_V_3_ increased intracellular Ca^2+^ and Mg^2+^ levels in K562 cells. Accumulating data also indicates that ROS have beneficial anti-cancer effects. For instance, increasing ROS levels can enhance apoptosis and thereby inhibit tumor growth [12]. Selenium can also induce the generation of ROS and apoptosis in tumor cells [13]. Moreover, Na_5_SeV_5_O_18_.H_20_, an analog of Se_2_Mo_10_V_3_, induced an increase in ROS levels in K562 cells [7]; here, Se_2_Mo_10_V_3_ also increased ROS levels in K562 cells. Efficient pH maintenance reduces apoptosis, suggesting that intracellular pH homeostasis and tumor development may be linked [14], and decreasing pH is a hallmark of apoptosis [7]. Consistent with these findings, we observed here that Se_2_Mo_10_V_3_ increased H^+^ levels, which corresponds to a decrease in pH, in K562 cells. During apoptosis, increasing mitochondrial permeability reduces Δ*φ*_m_ and triggers release of cytochrome C into the cytoplasm [15]. Here, Se_2_Mo_10_V_3_ decreased Δ*φ*_m_ and triggered the subsequent release of cytochrome C in K562 cells. Changes in the concentration of intracellular ions such as Ca^2+^ and Mg^2+^ may also induce mitochondrial impairment and reduce Δ*φ*_m_ [16]. It is therefore possible that, in Se_2_Mo_10_V_3_-treated K562 cells, decreased Δ*φ*_m_ and cytochrome C release were triggered in part by increased levels of Ca^2+^ and Mg^2+^. In conclusion, the results of this study together with previous findings indicate conclusively that Se_2_Mo_10_V_3_ induces the apoptosis of K562 cells.

To determine whether Se_2_Mo_10_V_3_-induced apoptosis is mediated by the NF-κB/IκBα pathway, we also examined changes in NF-κB and IκBα. NF-κB translocation is normally inhibited by IκB proteins that sequester it in the cytoplasm after they are phosphorylated by IκB kinase. Degradation of IκBα thus allows nuclear translocation of NF-κB [17, 18]. It has been reported that NF-κB/IκBα is constitutively active in breast, colon, pancreatic, ovarian, lymphoma, and melanoma cancer cells. Furthermore, inhibition of this pathway using chemotherapeutics may be able to reverse or halt the growth and spreading of tumors [19]. Selenium compounds also inhibit NF-κB in tumor cells [17]. Here, Se_2_Mo_10_V_3_ inhibited the degradation of IκBα and the translocation of NF-κB in K562 cells. Shishodia and colleagues demonstrated that translocated NF-κB activates the expression of anti-apoptotic genes such as Bcl-2 and Bcl-xL, and the suppression of NF-κB inhibited the expression of these genes, thereby promoting apoptosis [20]. Immunocytochemistry also revealed that Se_2_Mo_10_V_3_ treatment decreased Bcl-2 expression and increased Bax expression in K562 (data not shown). An elevated Bax/Bcl-2 ratio increases spontaneous self-oligomerization of those molecules, which facilitates the reduction of Δ*φ*_m_, the release of cytochrome C into the cytoplasm, and, ultimately, apoptosis [21]. Based on these findings, it is likely that decreased Δ*φ*_m_ and the release of cytochrome C in Se_2_Mo_10_V_3_-treated K562 cells are triggered in part by an elevated Bax/Bcl-2 ratio.

In summary, Se_2_Mo_10_V_3_ inhibits tumor growth both *in vitro* and *in vivo* and induces apoptosis in K562 cells at least in part by inhibiting NF-κB/IκBα. Our results indicate that Se_2_Mo_10_V_3_ may be a useful component of clinical therapy. Further research is needed to examine the anti-leukocythemia effects of Se_2_Mo_10_V_3_ *in vivo* and to evaluate its preclinical safety on animals.

## MATERIALS AND METHODS

### Drugs and chemicals

Se_2_Mo_10_V_3_ (yellow crystal, purity >99%) was synthesized by the State Key Laboratory of Applied Organic Chemistry, Lanzhou University, China. Briefly, solution 1 (0.29 g NH_4_VO_3_ dissolved in 10 mL hot water) and solution 2 (1.85 g (NH_4_)_6_Mo_7_O_24_·4H_2_O dissolved in 8 mL hot water) were blended and 0.063 g/mL Na_2_SeO_4_ were added while stirring (solution 3). Solution 3 was adjusted to a pH of 2 using H_2_SO_4_ (1:1), and its color changed from orange red to crimson (solution 4). Solution 4 was stirred with reflux at 90°C for 10 h (solution 5). Solution 5 was filtered while hot. The filtrate was placed at room temperature and the yellow crystals separated out quickly. After vacuum filtration, the crystal was washed using an aqueous solution (pH was adjusted to 2 using H_2_SO_4_ (1:1)) and dried. The weight of the crude product was 0.68g. The crude product was recrystallized 2 times using an aqueous solution (pH=2). 1.12g yellow crystal was obtained (Se_2_Mo_10_V_3_). The yellow crystal decomposed readily in the air and was slightly soluble in water, methanol, DMSO, and acetone [22]. RPMI-1640 nutrient solution was purchased from Invitrogen. Fetal bovine serum was purchased from Sijiqing Co. 3-(4,5-dimethylthiazol-2-yl)-2,5-diphenyl tetrazolium bromide (MTT) was purchased from Sigma.

### Cell culture

The human chronic myelogenous leukemia K562 cell line was supplied by Shanghai Cell Bank, Chinese Academy of Sciences and grown in RPMI-1640 nutrient solution supplemented with 10% fetal bovine serum, 2 mM L-glutamine, 100 units/mL penicillin, and 100 μg/mL streptomycin in a humidified 5% CO_2_ incubator at 37°C. The *in vitro* experiments were performed using a concentration of 1×10^5^ cells/mL.

### MTT assay

Cytotoxicity was measured in an MTT assay with slight modifications. K562 cells were treated with 12.5, 25, 50, or 100 mg/L Se_2_Mo_10_V_3_ for 24, 48, or 72 h. MTT solution was then added and the cells were incubated for an additional 4 h. The formazan crystals were dissolved with SDS solution and quantified using a microplate reader (ELx800, Bio-TEK). Cytotoxicity is presented as percent inhibition relative to the control cells. The half-maximal inhibitory concentrations (IC50) for Se_2_Mo_10_V_3_ in K562 cells were calculated using IC50 calculation software.

### Transmission electron microscopy (TEM)

K562 cell morphology was examined using TEM. Cells were treated with 50 mg/L Se_2_Mo_10_V_3_ for 24 h. Cell precipitates were then fixed with glutaraldehyde, post- fixed with osmium tetroxide, dehydrated with a graded alcohol series, immersed in epoxy resin and acetone, and embedded in epoxy resin. Ultra-thin sections were then prepared, dyed using uranyl acetate and lead citrate, and examined with a JEM-100 CX-II TEM (Jeol).

### Confocal laser scanning microscopy (CLSM)

Levels of Ca^2+^, Mg^2+^, H^+^, and reactive oxygen species (ROS) and mitochondrial membrane potential (Δ*φ*_m_) in K562 cells were determined by CLSM. Cells were treated with 12.5, 25, or 50 mg/L Se_2_Mo_10_V_3_ for 24 h. The cells were then incubated with Fluo-3/AM (5 μmol/L), Mag-fluo-4 (5 mmol/L), Carboxy SNARF-1/AM (10 μmol/L), 2′,7′-dichlorofluorescin diacetete D-399 (5 μg/mL), and Mito Tracker Green FM (1.25 μmol/L) (Molecular Probes, OR, USA) for 30-45 min at 37°C to determine the intracellular Ca^2+^, Mg^2+^, H^+^, and ROS levels and Δ*φ*_m_, respectively. Cells were then washed with RPMI-1640 medium and examined under a TCS SP2 CLSM (Leica, Germany). The results were analyzed with Leica Confocal software.

### Western blotting

Cytochrome C, IκBα, and NF-κB in K562 cells were detected by Western blotting. Cells were treated with 25, 50, or 100 mg/L Se_2_Mo_10_V_3_ for 24 h, resuspended in buffer A (10 mM Hepes-NaOH (pH 7.8), 15 mM KCl, 1 mM MgCl_2_, 0.1 mM EDTA, 1 mM DTT, 1 mM PMSF, 1 mg/L Leupeptin, and 1% NP-40), and incubated on ice for 30 min. The samples were then centrifuged at 12,000 ×g for 30 min at 4°C and the supernatants were used as cytoplasmic extracts. The remaining pellets were resuspended in buffer B (20 mM Hepes-NaOH (pH 7.9), 1.5 mM MgCl_2_, 0.42 M NaCl, 0.2 mM EDTA, 25% glycerol, 0.5 mM DTT, 0.5 mM PMSF, and 1 mg/L Leupeptin), incubated on ice for 30 min, and centrifuged at 14,000 ×g for 30 min at 4°C. The supernatants were used as nuclear extracts. All supernatants were quantified and run on 12% or 15% SDS polyacrylamide gels. Separated proteins were transferred from the gels to nitrocellulose membranes. The remaining procedures were performed according to the instructions provided with the LumiGLO Western blotting kit (KPL, MD, USA). Mouse monoclonal anti-cytochrome C (1:1000) (Invitrogen, CA, USA), rabbit monoclonal anti-IκBα (1:1000) (Abcam, MA, USA), and rabbit monoclonal anti-NF-κB (1:250) (Zymed Laboratory, CA, USA) were used as primary antibodies. Immunoblotting results were semi-quantified using Quantity One software (Bio-Rad).

### Animals and xenograft tumor experiments

5-week old female mice (species: Kun-Ming; strain: Swiss) weighing 20.0±2.0 g were purchased from the GLP Laboratory, Lanzhou University (Grade II, Certificate No. 14-005). They were housed at 23±1°C under 12h light/12h dark conditions with *ad libitum* access to food and water. All animal experiments were performed in accordance with relevant guidelines and regulations approved by the Experimental Animal Research Committee of Lanzhou University. According to standard xenograft tumor research protocols, mice were given subcutaneous injections of 3×10^6^ sarcoma 180 (S180) or hepatoma 22 (H22) cells in the exponential growth phase into the right axillary fossa on day 0. On day 1, mice were randomly divided into control, low, middle, and high dose groups and were treated daily with 0.9% normal saline or 1, 5, or 10 mg/kg Se_2_Mo_10_V_3_, i.g., respectively (n=10 per group). The mice were sacrificed on day 10 and the tumors were removed and weighed. Inhibition of tumor growth was evaluated using percent inhibition relative to tumor weights in control tumor-bearing mice.

### Statistical analysis

Data are shown as means ± S.D. of three independent experiments. Statistical analysis were performed using one-way ANOVA and the LSD method in SPSS 22.0 software. Differences were considered statistically significant when *P*<0.05.

## Abbreviations

Se_2_Mo_10_V_3_: (NH_4_)_2_H_15_Se_2_^VI^Mo_10_V_3_O_52_·2H_2_O
IC50: half-maximal inhibitory concentration
TEM: transmission electron microscope
Δ*φ*_m_: mitochondrial membrane potential
CLSM: confocallaser scanning microscopy
S180: sarcoma 180
H22: hepatoma 22
