# Correction: Se_2_Mo_10_V_3_, a heteropoly compound containing selenium, inhibits tumor growth

**DOI:** 10.18632/oncotarget.26408

**Published:** 2018-11-23

**Authors:** Hong-Ning Zhang, Wei-Li Feng, Chun-Na An, Wen-Guang Li

**Affiliations:** ^1^ Department of Pharmacology, School of Basic Medical Sciences, Capital Medical University, Beijing 100069, China; ^2^ Department of Pharmacology, School of Basic Medical Sciences, Qinghai University, Xining 810001, China; ^3^ Department of Pharmacology, School of Basic Medical Sciences, North China University of Science and Technology, Tangshan 063009, China; ^4^ Department of Pharmacology, School of Basic Medical Sciences, Lanzhou University, Lanzhou 730000, China

**This article has been corrected:** The correct information of the Author contributions and Funding is given below:

**Author contributions**

H.-N. Z. performed the experiments. W.-L. F. helped to perform the *in vitro* experiment. C.-N. A. wrote the manuscript, prepared the figures and supplied publication costs. W.-G. L. designed the project. All authors reviewed the manuscript.

**FUNDING**

This work was supported by Science and Technology Program of Beijing Municipal Education Commission (KM201810025002) and Health Science Key Program of Hebei Province (ZD20140029).

Original article: Oncotarget. 2017; 8:67871-67877. https://doi.org/10.18632/oncotarget.18909

